# Identification of the Splicing Factor *GmSR34b* as a Negative Regulator of Salt Stress Response in Soybean Through Transcriptome and Alternative Splicing Analysis

**DOI:** 10.3390/ijms262311648

**Published:** 2025-12-01

**Authors:** Jin-Bao Gu, Yin-Jie Cheng, Cong Li, Bai-Hong Zhang, Yu-Hang Zhang, Xiao-Yan Liang, Yang Li, Yan Lin

**Affiliations:** 1 Institute of Nanfan & Seed Industry, Guangdong Academy of Science, Guangzhou 510316, China; 2 Zhanjiang Research Center, Institute of Nanfan & Seed Industry, Guangdong Academy of Science, Zhanjiang 524300, China; 3 State Key Laboratory of Crop Stress Adaptation and Improvement, School of Life Sciences, Henan University, Kaifeng 475004, China

**Keywords:** alternative splicing, transcriptome, salt stress, soybean, *GmSR34b*, splicing factor

## Abstract

Soil salinity severely threatens soybean productivity worldwide. While transcriptional responses to salt stress are well-documented, the role of post-transcriptional regulation, particularly alternative splicing (AS), remains underexplored. This study combines physiological phenotyping, transcriptome-wide analysis, and molecular genetics to uncover the mechanisms behind the differences in salt tolerance between the salt-sensitive variety Huachun 6 (HC6) and the resistant variety Fiskeby III. Under salt stress, Fiskeby III exhibited superior survival rates and maintained ion homeostasis, as evidenced by a lower Na^+^/K^+^ ratio, compared with HC6. Transcriptomic and splicing analysis revealed extensive salt-induced alternative splicing reprogramming. Genes undergoing differential AS were enriched in pathways related to stress response, ion transport, and RNA splicing. Based on the overlap with both differentially expressed genes (DEG) and alternative splicing (DAS) genes under salt stress, a key splicing factor, *GmSR34b*, was identified as a central regulator of AS under salt stress. Under NaCl stress, the expression of *GmSR34b* in leaves peaked at 1 h and a salt stress-specific splicing variant was rapidly induced. A comparative analysis showed that the Fiskeby III cultivar prioritized maintenance of the full-length transcript during prolonged stress, whereas the HC6 cultivar accumulated higher levels of the splicing variant. This indicates differences in the regulation of alternative splicing between these two cultivars. Functional validation confirmed that overexpression of *GmSR34b* in soybean hairy roots inhibited salt tolerance. This study provides novel insights into the molecular mechanisms of salt tolerance in soybean, suggesting potential strategies for breeding resilient crops through the manipulation of splicing regulators.

## 1. Introduction

Salt stress is a major abiotic stress that severely constrains global crop productivity and causes substantial crop losses [1,2]. Soil salinity reduces water potential due to the excessive accumulation of sodium (Na^+^) and chloride (Cl^−^) ions, generating osmotic stress which consequently inhibits plant growth and development [3]; in particular, salinity causes crops to accumulate Na^+^ and Cl^−^ in their root cells, disrupting ionic balance and leading to cytological damage. Additionally, the increased accumulation of Na^+^ competes with the uptake of essential cations such as potassium (K^+^), calcium (Ca^2+^), and magnesium (Mg^2+^), resulting in ion dysregulation that disturbs metabolic homeostasis and undermines plant vitality [4,5]. Furthermore, salt stress triggers the excessive production of reactive oxygen species (ROS), including hydrogen peroxide and superoxide anions, which provoke oxidative stress and can have harmful effects on plants [6,7].

The responses of plants to salt stress involve complex physiological and molecular adaptations, including ion homeostasis maintenance, osmotic adjustment, and ROS scavenging [3,8,9]. Recent studies have revealed that transcriptional regulation plays a crucial role in salt stress responses, with numerous differentially expressed genes (DEGs) having been identified in various plant species under salinity stress [6,7]. However, increasing evidence suggests that post-transcriptional regulation, particularly alternative splicing (AS), represents another critical layer of gene expression control in response to environmental stresses [10,11,12]. AS leads to the generation of multiple mRNA isoforms from a single gene, dramatically expanding proteomic diversity and functional complexity. Alternative splicing has been found to occur with a frequency of 41–61% in plants, according to genome-wide transcriptome mapping studies [13,14,15]. This process includes five major types of alternative splicing: exon skipping (ES), alternative 5′ splice sites (A5SS), alternative 3′ splice sites (A3SS), intron retention (IR), and mutually exclusive exons (MXEs) [11]. For example, in cotton (*Gossypium davidsonii*), salt stress has been reported to induce 1287 and 1228 differential alternative splicing (DAS) events in roots and leaves, respectively, with IR being the most frequent AS type [16]. The regulatory significance of AS in stress responses extends beyond model plants to important crops. In cotton, 13 genes encoding SR proteins were shown to exhibit alternative splicing under salt stress, indicating a complex regulatory network [16]. Similarly, in date palm (*Phoenix dactylifera* L.), the pathway of the spliceosome was enriched in the category of upregulation under salt stress, highlighting the potential role of AS in its stress response [15]. These findings are consistent with the role of AS in fine-tuning stress-responsive pathways, such as the salt overly sensitive (SOS) pathway, which is involved in Na^+^/K^+^ homeostasis [11,13,15].

Serine/arginine-rich (SR) proteins, a conserved family of splicing factors, play pivotal roles in both constitutive and alternative splicing through the recognition of splice sites and regulation of spliceosome assembly [17,18]. These proteins typically contain one or two N-terminal RNA recognition motifs (RRMs) and a C-terminal domain rich in arginine–serine dipeptides (RS domain), which facilitate protein–protein and protein–RNA interactions [19,20,21]. Numerous studies have demonstrated that SR proteins undergo AS themselves and modulate the splicing of stress-responsive genes in response to various abiotic stresses [17]. For instance, in Arabidopsis, several SR proteins show stress-induced alternative splicing patterns that potentially alter their functional properties. Similarly, in date palm, salt stress triggers extensive AS events involving splicing factors such as *PdRS40*, *PdRSZ21*, and *PdSR45a*, suggesting conserved mechanisms across plant species [15]. The serine/arginine-rich splicing factor SC35 has been identified as a regulator of *bZIP49* mRNA splicing through a self-developed experimental method, and its overexpression increased salt sensitivity in *Populus tomentosa* [22]. In cassava (*Manihot esculenta*), SR proteins such as MeSCL33 modulate the AS of key genes involved in abscisic acid (ABA) biosynthesis, thereby influencing post-harvest physiological deterioration resistance through maintenance of ROS homeostasis [23]. However, the specific functions of SR proteins in soybean salt tolerance, particularly their genotype-dependent splicing patterns and regulatory mechanisms, remain poorly understood.

Soybean (*Glycine max*) is a critical legume crop providing high-quality protein and oil, which is particularly vulnerable to salt stress [24,25]. Soybean yields decreased by 52.5% at soil salinity levels of 14–15 dS/m and by 61.1% at levels of 18–20 dS/m [26]. Salt stress negatively affects nearly every stage of the soybean life cycle, hindering seed germination, seedling growth, and reproductive development by disrupting essential biological processes such as photosynthesis [27]. Using reverse genetics approaches, multiple genes associated with salt tolerance in soybean have been identified [28,29]; these include ion channel proteins such as the vacuolar Na^+^/H^+^ antiporter GmCHX1, which helps to sequester sodium in vacuoles, and transcription factors such as the membrane-bound GmNTL1, which regulates ion transport and genes related to reactive oxygen species [28,30]. *SST1*, a key salt tolerance gene which encodes a pentatricopeptide repeat (PPR) protein, has been identified through genome-wide association analysis [31]. Its truncated allele *SST1^HapT^*, containing a nonsense mutation, enhanced salt tolerance in soybean, while the full-length allele *SST1^HapC^* did not [31]. Despite these advances, due to the extensive size and genetic diversity of the soybean genome, numerous tolerance-related loci likely remain undiscovered [32,33,34].

In this study, we hypothesized that the differential salt sensitivity between the soybean varieties Fiskeby III and HC6 is regulated at the post-transcriptional level, particularly through alternative splicing (AS) events mediated by splicing factors such as *GmSR34b*. Our objectives were to (1) characterize the physiological and transcriptional responses of both varieties under salt stress, (2) identify key splicing factors involved in their salt stress responses, and (3) functionally validate the role of *GmSR34b* in salt resistance. To achieve these goals, we integrated phenotyping, transcriptome-wide analysis, and molecular genetics approaches. We expect our findings to provide novel insights into the role of AS in salt resistance, offering potential strategies for the molecular breeding of salt-resistant soybean varieties.

## 2. Results

### 2.1. Differential Salt Sensitivity of the Two Soybean Varieties

**Figure 1:**
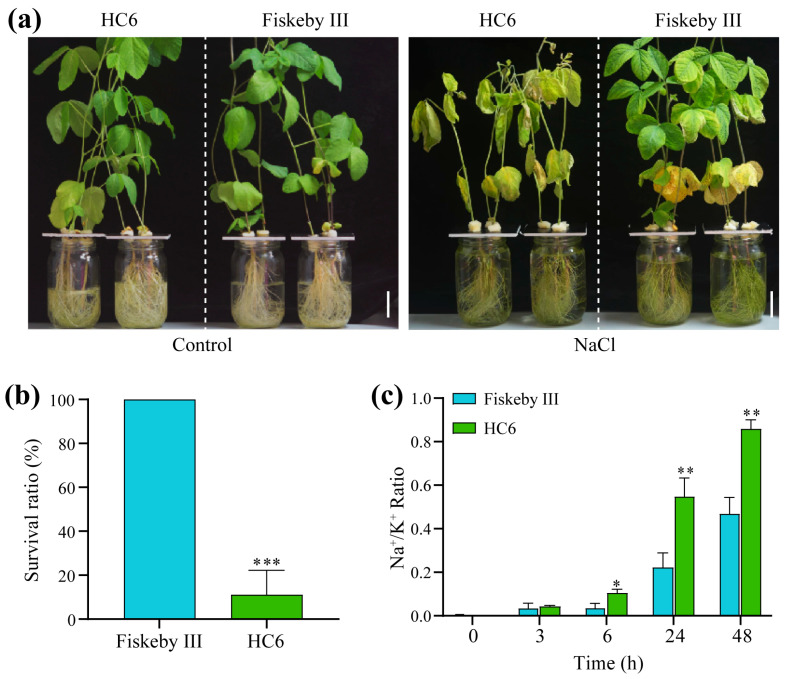
Salt sensitivity phenotypes and physiological responses of HC6 and Fiskeby III. (**a**) Phenotypic comparison of hydroponically grown seedlings under control (left) and 150 mM NaCl (right) conditions for 7 days. Scale bars: 2 cm. (**b**) Survival rates after 7 days of NaCl treatment (n = 6 biological replicates; *** *p* < 0.001, Student’s *t*-test). (**c**) Time-course analysis of Na^+^/K^+^ ratio in shoots under 150 mM NaCl (n = 5 biological replicates; * *p* < 0.05, ** *p* < 0.01, two-way ANOVA with Tukey’s post hoc test).

To investigate the difference in salt sensitivity between the considered soybean varieties (i.e., Glycine max cv. HC6 and Fiskeby III), we compared their phenotypic responses, survival rates, and Na^+^/K^+^ homeostasis in shoots under NaCl stress. Under control conditions, both varieties exhibited healthy growth with green leaves, well-developed leaves, and no visible stress symptoms. In contrast, after 7 days of treatment with NaCl, a noticeable divergence in phenotypic characteristics was observed. (Figure 1a). HC6 exhibited severe leaf chlorosis and wilting, indicating significant salt damage. In contrast, Fiskeby III maintained relatively healthy leaf turgor and green pigmentation, with only mild chlorosis observed in older leaves. Supporting these phenotypic observations, the survival rate of Fiskeby was approximately 100%, which was significantly higher than that of HC6, which had a survival rate of less than 20% under NaCl stress (Figure 1b). The changes in the Na^+^/K^+^ ratio in the shoots of Fiskeby III and HC6 during a 48-h salt stress treatment. Both varieties showed an increase in the Na^+^/K^+^ ratio over time, but the salt-sensitive HC6 experienced a more pronounced and sustained rise compared to the tolerant Fiskeby III. After 3 h NaCl treatment, there was no significant difference between the two varieties, with both maintaining low ratios. However, by 6 h, Huachun 6 displayed a statistically higher ratio than that of Fiskeby III. The difference became even more pronounced at 24 h, the ratio of HC6 was over three times that of Fiskeby III. After 48 h, HC 6 reached its peak ratio of around 0.9, while Fiskeby III remained relatively stable at approximately 0.5. These findings indicate that HC6 experienced a rapid loss of ion homeostasis, whereas Fiskeby III managed to maintain a balanced Na^+^/K^+^ ratio, likely contributing to its superior salt resistance. These results confirm that Fiskeby III has superior salt resistance at the whole-plant level, while HC6 is more sensitive to salt stress.

### 2.2. Transcriptome Underlies Differential Salt Sensitivity Between Soybean Varieties Fiskeby III and HC6

**Figure 2:**
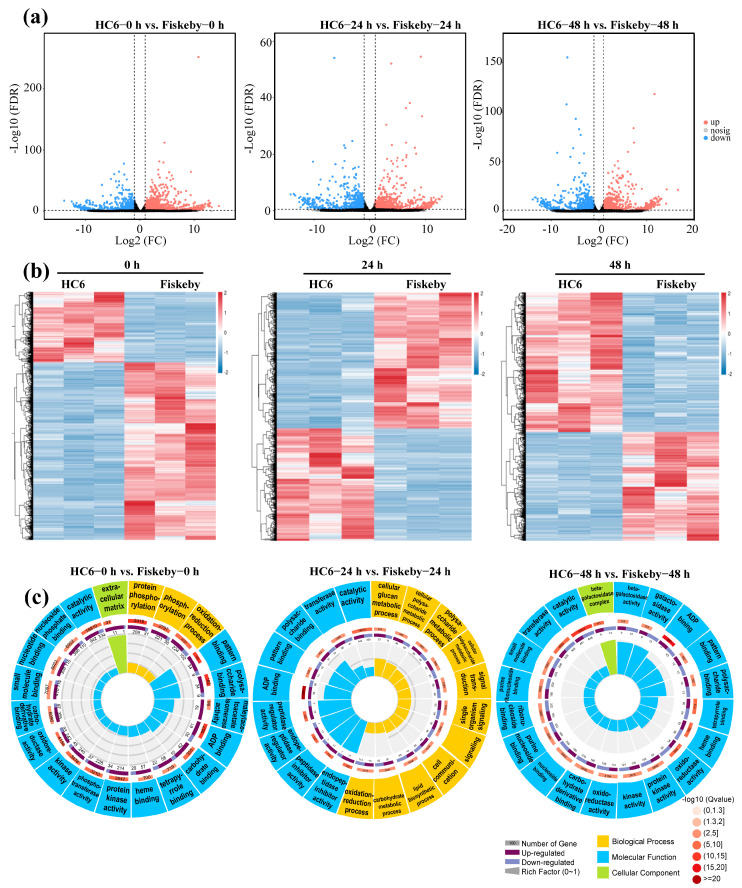
Transcriptome dynamics in leaves of Fiskeby III and Huachun 6 under salt stress. (**a**) Volcano plots showing DEGs between Fiskeby III and HC6 at 0, 24, and 48 h post-NaCl treatment (n = 3 biological replicates). Red: upregulated in Fiskeby III; blue: downregulated in Fiskeby III; gray: non-significant. Dashed lines indicate thresholds (|Log_2_FC| = 1, FDR = 0.05). (**b**) Heatmap of DEGs clustered by expression pattern (rows: genes; columns: samples). Color scale: Z-score of normalized expression (red means upregulated, blue means downregulated). (**c**) Circular GO enrichment plots of DEGs in the HC6 vs. Fiskeby III comparison. Colors represent GO categories (BP: biological process; MF: molecular function; CC: cellular component). The inner circle shows the number of DEGs and enrichment significance.

To elucidate the transcriptional basis of the observed differences in salt sensitivity, we compared the leaf transcriptomes of salt-tolerant Fiskeby III and salt-sensitive HC6 at 0, 24, and 48 h post-NaCl treatment. Principal Component Analysis (PCA) of transcriptomic data revealed that PC1 (74.1%) and PC2 (8.8%) explained 83% of the total transcriptional variation. At 0 h, salt-tolerant Fiskeby III and salt-sensitive HC6 formed distinct non-overlapping clusters, indicating inherent baseline differences. After the salt treatment, both genotypes shifted toward the positive PC1 axis, with the salt-sensitive HC6 exhibiting a more pronounced time-dependent response. In contrast, the tolerant Fiskeby III showed a milder response, with F-24h and F-48h clustering in the low-to-mid positive PC1 range. All replicates within each genotype–time group clustered tightly, confirming good reproducibility, and prolonged stress (48 h) exacerbated the separation between genotypes (Figure S1). Volcano plots revealed minimal basal differences under control conditions (0 h), with few genes exceeding significance thresholds (|Log_2_FC| > 1, FDR < 0.05) (Figure 2a; Table S2). However, salt stress triggered dynamic divergence: at 24 h, Fiskeby III exhibited more upregulated genes (red dots) while HC6 showed greater downregulation (blue dots); by 48 h, HC6 displayed extensive transcriptional repression with larger |Log_2_FC| magnitudes, whereas Fiskeby III maintained robust stress-responsive upregulation. Heatmap clustering confirmed distinct temporal patterns: Fiskeby III formed coordinated up/downregulated modules at 24/48 h, reflecting precise stress regulation, whereas HC6 showed homogeneous repression by 48 h with fewer activated clusters (Figure 2b).

Circular GO enrichment plots were constructed to highlight the functional divergence in DEGs. The analysis categorized DEGs (HC6 vs. Fiskeby III) into biological processes (BP, yellow), molecular functions (MF, blue), and cellular components (CC, green), with the outer ring indicating enrichment category and the inner ring showing the number of upregulated (dark purple) and downregulated (light purple) genes for each term. At 0 h (control), enrichment was limited to basal metabolic processes (e.g., oxidation-reduction, protein phosphorylation) and molecular functions (e.g., catalytic activity, binding). After 24 h of stress, stress-responsive biological processes (e.g., signal transduction, cell communication) and molecular functions (e.g., transferase activity, oxidoreductase activity) emerged as prominent, with Fiskeby III showing a greater number of up-regulated DEGs–indicating early adaptive transcriptional adjustments to stress (Figure 2c; Table S2). By 48 h, prolonged salt exposure drove strong, highly significant enrichment in carbohydrate metabolic processes (e.g., beta-galactoside metabolism) and cell wall remodeling (e.g., beta-galactosidase activity) in Fiskeby III, which maintained sustained up-regulation of these terms. In contrast, HC6 exhibited widespread down-regulation of metabolic and catalytic functions at 48 h, highlighting its inability to sustain adaptive responses under prolonged stress. These results underscore the contrasting transcriptional strategies underlying salt sensitivity: Fiskeby III activates protective mechanisms to mitigate stress, whereas HC6 undergoes irreversible stress-induced impairment.

### 2.3. Retained Intron (RI) and Skipped Exon (SE) Are the Predominant AS Types

**Figure 3:**
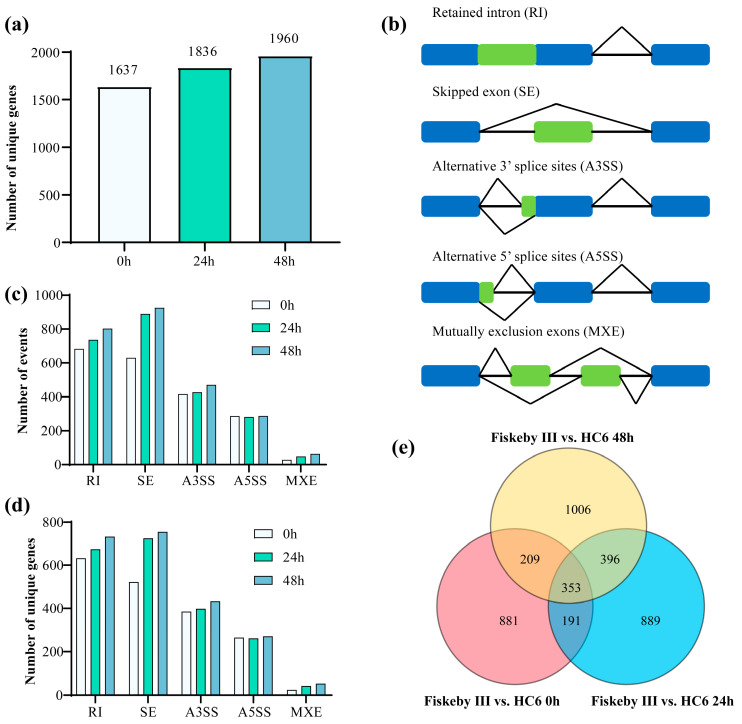
Alternative splicing dynamics under salt stress. (**a**) Number of unique genes with alternative splicing (AS) at 0, 24, and 48 h. (**b**) Schematic of five major AS types. (**c**) Distribution of AS events across types and time points. (**d**) Number of unique genes per AS type in Fiskeby III and HC6. (**e**) Venn diagram of AS genes between Fiskeby III and HC6 at 0, 24, 48 h time points, showing unique and overlapping genes.

Totals of 1637, 1836, and 1960 unique genes were identified to undergo AS at 0, 24, and 48 h, respectively, with the number of AS-related genes increasing over time (Figure 3a). Five major AS types were detected: retained intron (RI), skipped exon (SE), alternative 3′ splice site (A3SS), alternative 5′ splice site (A5SS), and mutually exclusive exons (MXEs) (Figure 3b). The total number of AS events increased progressively with the duration of salt stress, growing from 0 h to 24 h and then to 48 h, consistent with the upward trend observed in Figure 3a. Among the five types of splicing events, SE were the most abundant at all time points, peaking at 923 events at the 48-h mark and remaining the largest category at 48 h. RI and A3SS/A5SS followed closely, each contributing between 300 and 500 events at each time point. In contrast, MXE were the least common, with fewer than 200 events observed at any time point, suggesting a minimal role for this type in the response to salt stress (Figure 3c). Additionally, the number of unique genes also showed a time-dependent increase. Similar to the overall total of AS events, the unique genes involved in SE were the predominant type. RI and A3SS/A5SS were the next most prevalent unique types, while MXE remained insignificant (Figure 3d). These results suggest genotype-specific AS responses to salt stress.

The Venn diagram illustrates the unique AS genes found in Fiskeby III and HC6 under salt treatment at three different time points: 0, 24, and 48 h. Overlap analysis revealed that only 353 genes were shared among all three time points. In pairwise comparisons, the 24-h and 48-h groups exhibited the strongest similarity in AS profiles, with 396 shared events. This was followed by comparisons between the control group (CK) and the 48-h group, which had 333 shared events, and then between CK and the 24-h group, which showed 205 shared events. Additionally, each condition retained unique AS genes, with 881 in CK, 889 in 24 h, and 1006 in 48 h (Figure 3e). Overall, the diagram demonstrates that the duration of salt stress influences the extent and specificity of differential splicing between the resistant and sensitive varieties, with longer treatments resulting in a greater overlap of AS genes as well as a higher number of unique AS genes.

### 2.4. Identification of Stress-Responsive Splicing Factors Among SR Proteins

**Figure 4:**
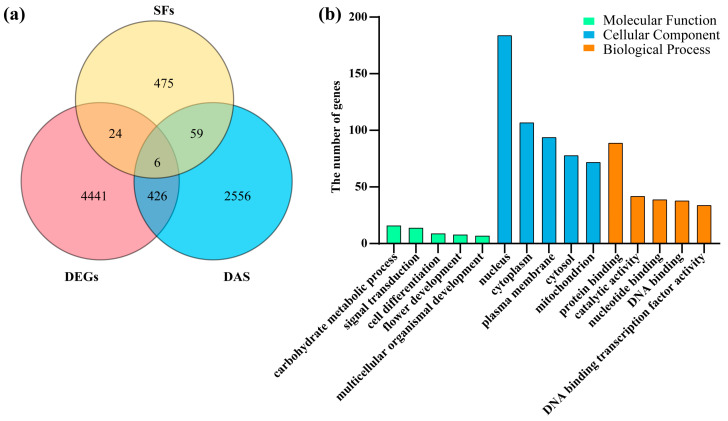
Integration of DEGs, DAS genes, and functional enrichment: (**a**) Venn diagram showing overlap between differentially expressed genes (DEGs), differentially alternatively spliced (DAS) genes, and splicing factors (SFs). Numbers indicate gene counts in each subset. (**b**) GO enrichment of the 515 shared SFs/DEG/DAS genes, categorized by biological process (BP), molecular function (MF), and cellular component (CC).

To analyze the interplay between transcriptional regulation and alternative splicing in salt stress responses, we integrated the results regarding DEGs and differentially alternatively spliced (DAS) genes and analyzed their functional enrichment. A three-way Venn diagram (Figure 4a) revealed limited overlap between DEGs, DAS genes, and splicing factors (SFs). Of the 4441 DEGs and 2556 DAS genes, only 515 genes were shared, indicating distinct regulatory pathways for transcriptional and post-transcriptional (splicing) responses to salt stress. Notably, only six genes overlapped between the DEGs, DAS genes, and SFs, suggesting minimal crosstalk between SF expression and their direct splicing targets under salt stress (Tables S3–S5).

GO enrichment analysis of the 515 shared SFs/DEG/DAS genes (Figure 4b) highlighted enrichment in cellular component (CC) terms, particularly those relating to the nucleus (including 185 genes) and cytoplasm (including 105 genes), reflecting subcellular compartments critical for stress signaling. For biological processes (BP), protein binding (including 92 genes) and catalytic activity (including 46 genes) were prominent, suggesting the roles of energy metabolism and protein interaction networks under stress. Molecular function (MF) terms were least represented, with only 20 genes enriched in the cytosol, indicating primary regulation at the functional and process levels rather than structural components.

### 2.5. Genotypic Variation in Splicing-Related Gene Expression and Molecular Characterization of GmSR34b Under Salt Stress

**Figure 5:**
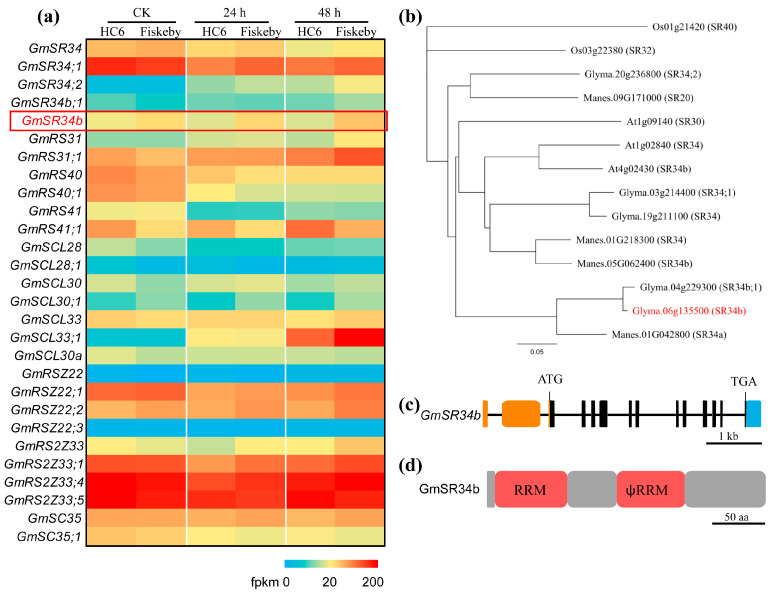
Characterization of splicing regulator *GmSR34b* in salt-stressed leaves. (**a**) Heatmap of splicing-related gene expression in Fiskeby III and HC6 under control (CK), 24 h, and 48 h NaCl treatment. Red box highlights *GmSR34b*. (**b**) Phylogenetic tree of SR proteins from soybean (Glyma), Arabidopsis (At), Cassava (Manes), and rice (Os). *GmSR34b* is marked in red. (**c**) Gene structure of *GmSR34b* (exons: black boxes; introns: lines; UTRs: orange/blue boxes). (**d**) Protein domain architecture of *GmSR34b* (RRM: red; ψRRM: pink). Scale bars: (**c**) 1 kb and (**d**) 50 amino acids (aa).

Considering the six genes overlapping between DEGs, DAS genes, and SFs, we focused on the expression patterns of splicing-related genes—particularly serine/arginine-rich (SR) proteins and SFs—in Fiskeby III and HC6 leaves under salt stress. The heatmap revealed genotype-specific and temporal regulation of these genes. *GmSR34b* (red box) was constitutively expressed at higher levels in Fiskeby III across all time points (CK, 24 h, 48 h) compared with HC6, where its expression declined at 48 h (Figure 5a). Notably, its splice variant *GmSR34b*:1 showed stress-induced upregulation in Fiskeby III (24–48 h) but downregulation in HC6. Other SR genes, such as *GmSR31* and *GmRS40*, exhibited divergent splicing isoform expression: Fiskeby III favored isoforms with prolonged upregulation, while HC6 isoforms showed transient induction followed by repression.

Phylogenetic tree analysis was conducted to classify the evolutionary relationships of 14 SR34 proteins based on amino acid sequences from various plant species, including *Glycine max* (soybean), *Arabidopsis thaliana* (Arabidopsis, At), *Manihot esculenta* (cassava, Manes), and *Oryza sativa* (rice, Os). The construction of the phylogenetic tree grouped *GmSR34b* (Glyma.06g135500) with *GmSR34b*;1 (Glyma.04g229300) and SR34b (Manes 05G062400) within a conserved clade. Importantly, this clade is separate from those containing the SR40 (Os01g21420) and SR32 (Os03g22380) homologs, which show longer branch lengths and a greater genetic distance from the *GmSR34b* group, indicating functional specialization. Structural analysis revealed that *GmSR34b* contains 12 exons and 11 introns. In the schematic, exons are represented by black boxes, introns by lines, and UTRs by orange and blue boxes (Figure 5c). The encoded protein (Figure 5d) harbors two conserved RNA recognition motifs (RRMs) and a pseudo-RRM (ψRRM)—domains that are critical for pre-mRNA binding and splicing site selection, consistent with its predicted role as a splicing factor.

### 2.6. GmSR34b Enhances Salt Sensitivity in Soybean Roots Through Stress-Induced Alternative Splicing and Genotype-Specific Expression

**Figure 6:**
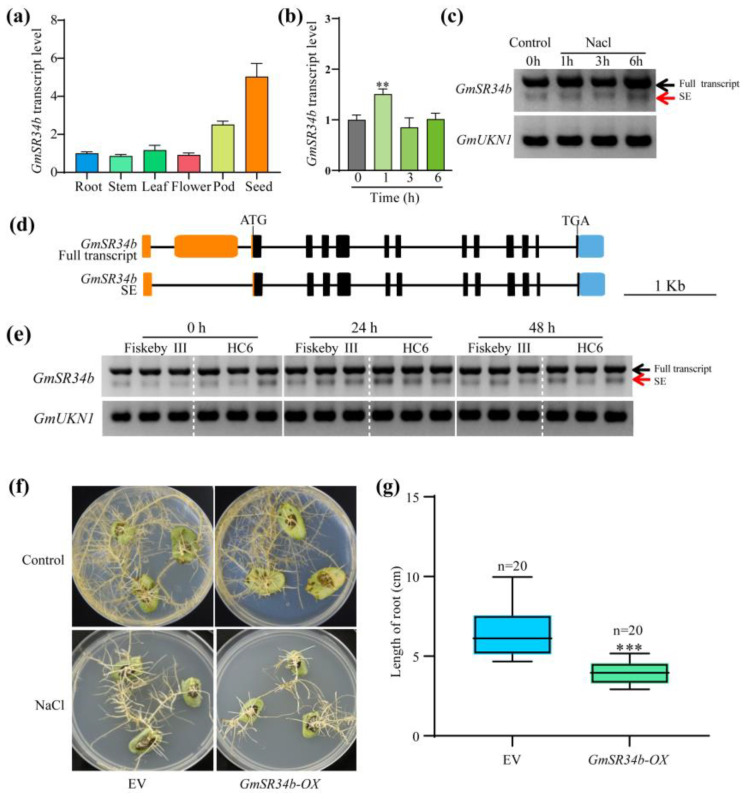
Functional characterization of *GmSR34b* in the context of salt tolerance. (**a**) Tissue-specific expression of *GmSR34b* (n = 5). (**b**) Time course expression under NaCl stress (** *p* < 0.01, Student’s t-test). (**c**) RT-PCR of *GmSR34b* transcripts (Full transcript: full-length; SE: skipped exon variant). (**d**) Schematic of *GmSR34b* gene structure (orange: 5′UTR; blue: 3′UTR; black boxes: exons). (**e**) Genotype-specific splicing dynamics in Fiskeby III vs. HC6. (**f**) Phenotype of EV and *GmSR34b-OX* hairy roots under control/NaCl (scale bars: 1 cm). (**g**) Root length quantification (*** *p* < 0.001, n = 20). *GmUKN1*: internal control.

Quantitative analysis revealed that *GmSR34b* was most highly expressed in seeds of HC6, with transcript levels 8.2-fold higher than in leaves (Figure 6a). Under NaCl stress, *GmSR34b* expression in leaves peaked at 1 h, followed by a decline at 3 h in the HC6 variety (Figure 6b), suggesting rapid stress-responsive induction. RT-PCR analysis identified two *GmSR34b* transcripts in both Fiskeby III and HC6: the full-length isoform (Full transcript) and a skipped exon variant (SE), resulting from the exclusion of the 5′UTR 2 (Figure 6d). The SE variant in HC6 was weak under control conditions but accumulated transiently at 1–3 h post-NaCl, indicating salt-induced alternative splicing (Figure 6c,d).

Comparative RT-PCR showed that Fiskeby III maintained higher levels of the full-length *GmSR34b* transcript at 24–48 h under salt stress, whereas HC6 exhibited reduced levels of the full-length transcript and increased SE variant accumulation at 48 h (Figure 6e). This divergence suggests that the genotype-specific differences in splicing patterns at the transcriptional level result in the production of more functional *GmSR34b* transcripts in Fiskeby III, potentially contributing to its superior stress tolerance compared to HC6.

Overexpression of *GmSR34b* (*GmSR34b*-OX) in soybean hairy roots significantly inhibited salt resistance compared with empty vector (EV) controls (Figure 6f). Under NaCl treatment, *GmSR34b*-OX roots showed 43% shorter root lengths and reduced root browning, indicating enhanced growth and reduced cellular damage (Figure 6g). Its overexpression exacerbates salt sensitivity, while genotype-dependent splicing dynamics may contribute to differential salt sensitivity between soybean genotypes. These findings highlight *GmSR34b* as a candidate for studying post-transcriptional regulation in plant salinity responses.

## 3. Discussion

Soil salinity is a major abiotic stress limiting crop productivity, affecting over 800 million hectares of arable land globally [35,36,37]. Soybean, a key staple and oilseed crop, is particularly sensitive to salt stress, with yield losses exceeding 40% under moderate salinity [38,39,40]. To mitigate these losses, identifying salt-responsive genes and unraveling their regulatory mechanisms are critical for developing tolerant cultivars [39,40]. AS, which is a post-transcriptional regulatory process that generates multiple mRNA isoforms from a single gene, has emerged as a key player in plant stress responses [41,42]. SR proteins are a family of conserved splicing factors, which modulate AS by recognizing cis-acting elements in pre-mRNAs and recruiting spliceosomal complexes [43,44,45]. In this study, we characterized the SR gene *GmSR34b* in soybean, revealing its salt-responsive expression, stress-induced alternative splicing, genotype-dependent regulation, and functional role in salt sensitivity. These findings provide new insights into the post-transcriptional mechanisms underlying plant salinity adaptation in leaves, as the primary photosynthetic organ and a critical site for stress-induced physiological dysfunction [31].

### 3.1. Transcriptome Analysis Under Salt Stress and AS Responses to Salt Stress in Soybean

Salt stress induced extensive transcriptional changes in both soybean varieties, with Fiskeby III exhibiting more robust and coordinated upregulation of stress-responsive pathways, including those related to ion transport, ROS scavenging, and ABA signaling (Figure 2a). In contrast, HC6 showed pronounced transcriptional repression and enrichment in pathways associated with cellular damage, such as catalytic activity and cellular glucan metabolic process (Figure 2b,c). These results highlight the fundamental role of transcriptional regulation in salt resistance.

Our findings reveal that the superior salt resistance of Fiskeby III in comparison with HC6 is not only attributed to differential gene expression but is substantially governed by post-transcriptional mechanisms, particularly AS (Figure 3a). Notably, AS events were overwhelmingly prevalent under salt stress, with RI and SE being the most abundant types (Figure 3c,d). The number of AS genes increased over time, indicating dynamic regulation of splicing with stress progression. Importantly, the AS landscape differed significantly between genotypes; that is, Fiskeby III and HC6 exhibited distinct sets of AS genes and divergent splicing patterns for shared genes (Figure 3e). This genotype-specific AS regulation suggests that splicing diversity may contribute to phenotypic variation in salt stress sensitivity.

### 3.2. Transcriptional and Alternative Splicing Landscapes Underlying Soybean Salt Stress Adaptation

Our integrated analysis of DEGs and DAS genes under salt stress reveals a complex but largely segmented regulatory landscape in soybean. The limited overlap between DEGs and DAS genes—only 432 overlapping out of a total of 4441 DEGs and 2556 DAS genes—suggests that transcriptional and post-transcriptional (splicing) responses to salt stress operate through predominantly independent mechanisms (Figure 4a). Notably, the minimal triple overlap—with just six genes shared between DEGs, DAS genes, and splicing factors (SFs)—implies that the activity of SFs may be regulated post-transcriptionally or through modifications other than changes in their own transcript abundance. This points to a possible layer of post-translational control governing splicing regulation under stress conditions. Functional enrichment analysis of the shared DEG/DAS genes further highlighted their roles in stress-responsive signaling and cellular organization. The strong enrichment observed in nuclear and plasma membrane locations underscores the significance of compartmentalized signaling and transcriptional regulation. Additionally, the prominence of these genes in mitochondrial organization and protein binding suggests the involvement of reprogramming of energy metabolism and stress-related protein interaction networks. The under-representation of cellular component terms reinforces the idea that responses to salt stress are primarily driven by dynamic molecular functions and biological processes, rather than static structural changes (Figure 4b). Among these candidates, *GmSR34b*, which is a gene that encodes a serine/arginine-rich protein, emerges as an intriguing target for future functional studies due to its potential dual role in both expression and splicing regulation.

### 3.3. Tissue-Specific Expression of GmSR34b and Biological Implications

The spatiotemporal expression pattern of *GmSR34b* suggests context-dependent functional roles in soybean (Figure 6a). High transcript levels in seeds and pods align with previous reports that SR proteins are enriched in reproductive tissues, where precise splicing regulation is critical for seed development and maturation [46,47,48]; for instance, Arabidopsis SR34 is required for pollen viability and seed set, while rice *OsSR45* regulates grain filling under heat stress [47,48,49]. The moderate expression of *GmSR34b* in leaves suggests additional roles in vegetative stress responses, consistent with our observation that *GmSR34b* was induced in leaves by salt stress (Figure 6b). Leaves are central to plant productivity under stress, with photosynthetic impairment and oxidative damage in leaf tissues directly reducing yield [50,51]. The rapid upregulation of *GmSR34b* in leaves at 1 h post-salinity aligns with the timeline of chloroplast ROS accumulation and photosynthetic decline—two early events in leaf salt stress [52]. This suggests that *GmSR34b* may act as a “first responder” to salinity in leaves, modulating splicing of downstream stress-responsive genes. Similarly, Arabidopsis SR30 is induced by ABA and salt, and its knockout altered the splicing of stress-related genes such as *RD22* (*Responsive to Dehydration*) and *KIN1* [53]. Similarly, *GmSR34b* may regulate the splicing of stress genes in soybean leaves, such as chloroplast-localized ROS scavengers or PSII repair factors to maintain fine-tuned photoprotection under saline conditions.

### 3.4. Salt-Induced Alternative Splicing of GmSR34b

AS of SR genes is a conserved regulatory mechanism that generates functionally distinct isoforms, often in response to stresses [54]. We detected two *GmSR34b* transcripts: a full-length isoform and a SE spliced variant with exon skipping (Figure 6c,d). The SE variant was specifically induced by salt stress in leaves (Figure 6c), echoing findings in Arabidopsis, where SR34 was shown to produce stress-specific AS isoforms with truncated RNA-binding domains [55]. Salt stress may disrupt the transcription of *GmSR34b* by increasing the production of SE isoforms, representing a negative feedback loop that dampens splicing activity.

The timing of SE induction (1–6 h post-salinity) coincides with the peak of *GmSR34b* transcriptional upregulation (1 h, Figure 6b,c), suggesting coordination between transcription and splicing. This “co-transcriptional” regulation is common for SR genes, where stress signals trigger both increased transcription and AS of SR pre-mRNAs, enabling rapid adjustment of the activity of splicing machinery [43]. This is particularly relevant for leaves, where precise control of the abundance of splicing factors is critical to avoid dysregulation of photosynthetic gene expression.

### 3.5. Genotype-Dependent Splicing Dynamics: Linking GmSR34b to Salt Tolerance

The salt-resistant genotype Fiskeby III and sensitive genotype HC6 exhibited striking differences in *GmSR34b* expression and splicing in leaves (Figure 6e). At baseline (0 h), Fiskeby III had higher full-length *GmSR34b* transcript levels than HC6, suggesting a pre-adaptive mechanism that primes the tolerant genotype for stress. Under salinity, HC6 showed a stronger induction of the SE isoform. This genotype-specific splicing bias may contribute to differential salt tolerance by altering the transcription of *GmSR34b*-regulated splicing networks in leaves. In Arabidopsis, the natural variation in AS of stress-responsive genes is associated with stress tolerance [43,56,57,58,59]; for example, accessions with salt-tolerant phenotypes showed constitutive splicing of SOS1 pre-mRNA, whereas sensitive accessions produced non-functional splice variants under saline conditions [60,61]. Similarly, the higher full-length *GmSR34b* levels in Fiskeby III leaves may ensure efficient splicing of essential salt-responsive genes, while the increase in SE isoforms in HC6 may disrupt splicing fidelity, leading to misregulation of downstream targets. This hypothesis is supported by our PCA results (previous data), which demonstrated that HC6 undergoes more transcriptional reprogramming in leaves under salt stress, potentially due to impaired splicing regulation.

Notably, the divergence in *GmSR34b* splicing between genotypes was amplified at 48 h (Figure 6e), aligning with the prolonged stress phase in which chloroplast degradation and photosynthetic collapse occur in sensitive genotypes [62]. This suggests that the splicing dynamics of *GmSR34b* in leaves may serve as a molecular timer for stress adaptation: tolerant genotypes maintain functional splicing machinery longer, preserving photosynthetic integrity, whereas sensitive genotypes lose this capacity, exacerbating leaf senescence.

### 3.6. GmSR34b Overexpression Inhibits Salt Tolerance in Soybean

To directly test the role of *GmSR34b* in salt tolerance, we generated overexpression (OX) lines and observed that *GmSR34b*-OX plants exhibited shorter root growth under salt stress compared with empty vector (EV) controls (Figure 6f,g). This phenotype indicates that *GmSR34b* acts as a negative regulator of salt tolerance. The negative role of *GmSR34b* may stem from its splicing of pro-stress genes or inhibition of tolerance-related splicing events; for instance, if *GmSR34b* promotes splicing of a repressor of the chloroplast antioxidant pathway, its overexpression would impair ROS scavenging, leading to PSII damage and leaf photoinhibition. Alternatively, *GmSR34b* may sequester other splicing factors, disrupting their function in stress responses—a phenomenon known as “splicing factor titration” [63,64]. The salt sensitivity of OX lines could also result from altered ratios of full-length to SE isoforms in leaves, as overexpression may saturate the splicing machinery and favor production of non-functional SE variants, leading to a dominant-negative effect. This is consistent with our observation that OX lines had higher SE isoform levels under salinity (Figure 6g), which may further exacerbate splicing dysregulation.

### 3.7. Implications for Soybean Salt Tolerance Breeding

Our findings highlight *GmSR34b* as a potential target for improving soybean salt tolerance via splicing engineering in leaves. The genotype-dependent splicing pattern (i.e., full-length enrichment in Fiskeby III) suggests that selecting for alleles with reduced SE isoform induction could enhance stress resilience. CRISPR-Cas9-mediated editing of *GmSR34b* splice sites to block SE production in leaves, or overexpression of the full-length isoform under a leaf-specific stress-inducible promoter, may mitigate salt-induced photosynthetic decline. Additionally, identifying downstream targets of *GmSR34b* in leaves could reveal novel splicing events underlying chloroplast protection, providing a multilevel regulatory network for breeding. Moreover, we acknowledge that further experiments specifically targeting germination and early shoot growth would be valuable to fully understand stage-specific tolerance mechanisms. Such work could include transcriptomic and splicing analyses of germinating seeds under salt stress, functional studies of germination-related genes affected by splicing, and comparative phenotyping of salinity-related effects across developmental stages.

## 4. Materials and Methods

### 4.1. Plant Materials and Growth Conditions

The soybean varieties Fiskeby III (salt-resistant) and Huachun 6 (HC6, salt-sensitive) were used in this study. Seeds were surface-sterilized with 75% ethanol for 30 s, rinsed with sterile water, and germinated on moist filter paper at 25 °C in the dark for 3 days. Uniform 12-day-old seedlings were transferred to hydroponic systems containing half-strength Hoagland’s solution (pH 5.8), either without (control) or with 150 mM NaCl, and grown in a growth chamber for 7 days (16 h light/8 h dark, 25 °C), and the shoots were harvested for Na^+^ and K^+^ concentrations analysis. The salt sensitivities of Fiskeby III and HC6 plants were evaluated based on their survival rate, leaf chlorosis, wilting severity, and Na^+^/K^+^ ratio under 150 mM NaCl stress.

For salt stress treatment, 150 mM NaCl was added to the hydroponic 1/2 Hogland solution at the three-leaf stage of HC6 and Fiskeby III, and leaves were sampled at 0, 1, 3, 6, 24, and 48 h post-treatment (hpt). Samples were immediately frozen in liquid nitrogen and stored at −80 °C for RNA extraction.

### 4.2. Measurement of Na^+^ and K^+^ Concentrations

The concentrations of Na^+^ and K^+^ in the digested samples were quantitatively determined using Inductively Coupled Plasma Optical Emission Spectrometry (ICP-OES). The instrument was calibrated with a series of multi-element standard solutions covering the expected concentration ranges for Na and K. The operating parameters for ICP-OES were set as follows: RF power, 1500 W; plasma gas (Ar) flow, 12 L min^−1^; auxiliary gas flow, 0.8 L min^−1^; nebulizer gas flow, 0.75 L min^−1^; nebulizer pressure, 2.8 bar; axial viewing mode; and replicate reading time, 3 s per replicate. The emission wavelengths used for detection were 589.592 nm for Na and 766.490 nm for K. Each sample was measured in triplicate. The ion concentrations were calculated based on the standard curves and expressed as milligrams per gram of dry weight (mg g^−1^ DW).

### 4.3. RNA Extraction and Quantitative Real-Time PCR

Total RNA was isolated from HC6 leaves using TRIzol^®^ reagent (Invitrogen, Carlsbad, CA, USA), following the manufacturer’s protocol. RNA quality was assessed via 1% agarose gel electrophoresis and a NanoDrop 2000 spectrophotometer (Thermo Fisher Scientific, Waltham, MA, USA). First-strand cDNA was synthesized from 2 μg of total RNA using the PrimeScript™ RT Reagent Kit with gDNA Eraser (Takara, Dalian, China). qRT-PCR was performed on a CFX96 Real-Time PCR System (Bio-Rad, Hercules, CA, USA) using SYBR^®^ Premix Ex Taq™ II (Takara, Dalian, China). The relative expression level of *GmSR34b* was calculated using the 2^−ΔΔCt method, with *GmEF-1α* (*Glyma.17g056000*) as the internal reference gene. Primers are listed in Supplementary Table S1.

### 4.4. Transcriptome Sequencing and Alternative Splicing Analysis

Total RNA was extracted from leaves RNA of Fiskeby III and HC6 at 0, 24, and 48 hpt using the Trizol reagent kit (Invitrogen, Carlsbad, CA, USA), according to the manufacturer’s protocol. RNA quality was assessed on an Agilent 2100 Bioanalyzer (Agilent Technologies, Palo Alto, CA, USA) and checked using RNase-free agarose gel electrophoresis. After total RNA was extracted, eukaryotic mRNA was enriched with Oligo(dT) beads. Then, the enriched mRNA was fragmented into short fragments using fragmentation buffer and reverse-transcribed to cDNA using the NEBNext Ultra RNA Library Prep Kit for Illumina (NEB #7530, New England Biolabs, Ipswich, MA, USA). The resulting cDNA library was sequenced using an Illumina Novaseq6000 by Gene Denovo Biotechnology Co. (Guangzhou, China). Raw reads were filtered using fastp (version 0.18.0) to remove adapters and low-quality sequences. Clean reads were mapped to the soybean reference genome (Wm82.a2.v1) using HISAT2 v2.2.1. DEGs was analyzed using DESeq2 v1.34.0, with thresholds of |log_2_(fold change)| > 1 and FDR < 0.05. The software rMATS (version 4.0.1) (http://rnaseq-mats.sourceforge.net/index.html, accessed on 15 March 2022) was used to identify alternative splicing events and analyze differential alternative splicing events between samples. We identified AS events with a false discovery rate (FDR) < 0.05 in a comparison as significant AS events.

### 4.5. Phylogenetic and Structural Analysis of GmSR34b

The coding sequence (CDS) and amino acid sequence of *GmSR34b* (Glyma.06g135500) were retrieved from Phytozome v14 (https://phytozome.jgi.doe.gov/, accessed on 12 July 2025). Homologous sequences from Arabidopsis, rice and cassava were obtained via BLASTP searches against the NCBI database. A phylogenetic tree was constructed using MEGA 7.4 with the neighbor-joining method (1000 bootstrap replicates). The gene structure of *GmSR34b* was visualized using GSDS 2.0 (http://gsds.gao-lab.org/, accessed on 10 July 2025), and protein domains were predicted via SMART (http://smart.embl-heidelberg.de/, accessed on 10 July 2025), highlighting conserved domains.

### 4.6. Cloning and Vector Construction of GmSR34b

Total RNA from HC6 leaf was reverse-transcribed to cDNA. The full-length CDS of *GmSR34b* was amplified using gene-specific primers (Supplementary Table S1) and cloned into the binary vector pCAMBIA1300 under the control of the CaMV 35S promoter. The recombinant plasmid (35S::*GmSR34b*) was verified via Sanger sequencing and transformed into Agrobacterium rhizogenes strain K599 for hairy root transformation.

### 4.7. Hairy Root Transformation and Salt Tolerance Assay

Soybean cotyledons (7-day-old seedlings) were infected with A. rhizogenes K599 carrying 35S::*GmSR34b* (overexpression, OX) or empty vector (EV, control). Infected cotyledons were cultured on 1/2 MS medium (0.8% agar, 3% sucrose, pH 5.8) in the dark for 2 weeks to induce hairy root formation. Transgenic roots were identified via PCR using bar gene primers (Supplementary Table S1). For salt tolerance assays, EV and OX hairy roots were transferred to 1/2 MS medium with or without 150 mM NaCl and grown vertically for 7 days. Root length was measured using ImageJ v1.8.0, and phenotypes were documented with a digital camera.

### 4.8. RT-PCR and Splicing Variant Analysis

To analysis the transcript levels of *GmSR34b* in different organs, RT-PCR was performed using cDNA from root, stem, leaf, flower and pod in HC6 with primers (Supplementary Table S1). Short-term expression dynamics of *GmSR34b* in HC6 leaves treated with 150 mM NaCl were analyzed over a 6-h time course.

To detect *GmSR34b* splicing variants, RT-PCR was performed using cDNA from Fiskeby III and HC6 leaves under 150 mM NaCl treatment (0, 24, 48 hpt) with variant-specific primers (Supplementary Table S1). PCR products were separated on 1.5% agarose gels and visualized with ethidium bromide.

### 4.9. Statistical Analysis

All experiments were performed with at least three biological replicates. Data are presented as mean ± standard deviation (SD). Significant differences were determined via Student’s t-test or one-way ANOVA with Tukey’s post hoc test (*p* < 0.05, *p* < 0.01, or *p* < 0.001) using GraphPad Prism v9.0.

## 5. Conclusions

In summary, our data demonstrate that *GmSR34b* is a salt-responsive SR gene with tissue-specific expression, salt-induced alternative splicing, and genotype-dependent regulation in leaves. Its overexpression repressed salt tolerance by inhibiting root growth, suggesting a negative role in salt tolerance. The differential splicing dynamics between tolerant and sensitive genotypes highlight AS as a key mechanism underlying salt adaptation in soybean. These findings advance our understanding of post-transcriptional regulation in plant salt responses and provide a foundation for developing salt-tolerant soybean varieties through the engineering of splicing variants, specifically targeting leaf function.

## Data Availability

All data supporting the conclusions of this study are included in the main text and the Supplementary Materials.

## Supplementary Materials

The following supporting information can be downloaded from https://www.mdpi.com/article/10.3390/ijms262311648/s1.
