# Supplementary material for: Identification of the Splicing Factor GmSR34b as a Negative Regulator of Salt Stress Response in Soybean Through Transcriptome and Alternative Splicing Analysis
Source: Int J Mol Sci. 2025 Dec 1;26(23):11648. doi: 10.3390/ijms262311648 (PMC12692361; doi:10.3390/ijms262311648)
Supplement: Supplementary file 1 [file ijms-26-11648-s001.zip › Figure S1.pdf]

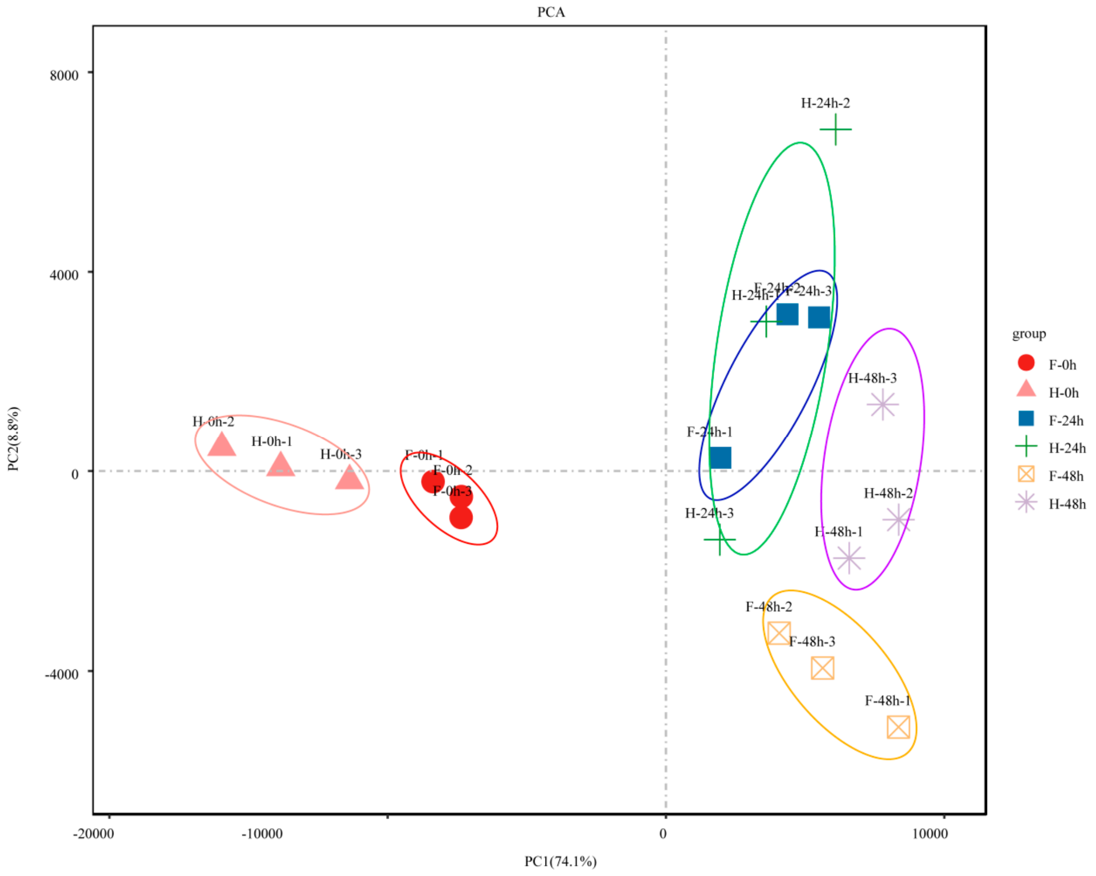


**Figure S1.** PCA analysis of transcriptome profiles under salt stress.Principal Component Analysis (PCA) of RNA-seq data showing global transcriptional variation among samples. The x-axis represents PC1 (74.1% variance explained), and the y-axis represents PC2 (8.8% variance explained). Samples are grouped by genotype and time point:Fiskeby III (tolerant): F-0h (red circles), F-24h (blue squares), F-48h (orange diamonds). HC6 (sensitive): H-0h (pink triangles), H-24h (green crosses), H-48h (purple stars). Biological replicates (n=3) are labeled as "-1/-2/-3". Ellipses indicate 95% confidence intervals for each group. Salt stress (150 mM NaCl) induces distinct transcriptional reprogramming between genotypes, with Fiskeby III showing a more coordinated response at 24h and 48h compared to HC6.
